# Statins; the *panacea* of cardiovascular disease

**DOI:** 10.1007/s12471-017-0967-2

**Published:** 2017-03-06

**Authors:** G. A. de Waard, N. van Royen

**Affiliations:** 1 Department of Cardiology, VU University Medical Center, Amsterdam, The Netherlands; 2 International Centre for Circulatory Health, Imperial College London, London, UK

In this issue of the Netherlands Heart Journal with a focus on *Atherosclerosis*, Cerit and colleagues investigate the impact of statin use on the corrected thrombolysis myocardial infarction frame count (CTFC) after elective percutaneous coronary intervention (PCI) [1]. CTFC counts the number of frames required for contrast material to reach a standardised distal landmark and thereby serves as a surrogate of coronary blood flow velocity. In this retrospective study, the authors divide a population of 80 consecutive patients with stable ischaemic heart disease into two groups based on the use of statins prior to PCI. The study is observational and neither randomisation nor propensity score matching has been applied to mitigate bias. Nevertheless, the baseline variables of the two groups are well balanced.

In both the statin and control group, CTFC decreased (coronary flow improved) substantially after PCI. As no pharmacological agents were given to invoke coronary hyperaemia, this observation in itself is interesting and merits further exploration. Myocardial perfusion is regulated tightly through myocardial, metabolic and endothelium-based processes, collectively termed autoregulation [2]. Perfusion is maintained at a stable plateau in the structurally normal heart under resting conditions. The mechanism of autoregulation can compensate for the presence of a stenosis that would otherwise limit coronary flow [2]. Only in case of very tight lesions (>85% stenosis as measured by quantitative coronary angiography), does the autoregulatory compensation mechanism fall short and is coronary flow limited under resting conditions (although well-developed collateral arteries can maintain sufficient myocardial perfusion). For this reason, true resting coronary flow typically remains unchanged after PCI. Exercise or vasodilators, such as adenosine, that increase coronary flow are needed to exhaust the autoregulatory compensation mechanism and detect an improvement of maximal flow by PCI. In the studied population, however, CTFC improved after PCI irrespective of statin prescription. This could be explained by two factors. Firstly, stenosis diameters were, on average, 80 and 85% in the non-statin and statin groups, respectively. This suggests that in a proportion of the studied population, stenosis diameter exceeded 85% and autoregulation was exhausted, leading to reduced resting flow before PCI [3]. Secondly, there may have been a pseudo-hyperaemic state after PCI. Contrast medium itself acts as a vasodilator and interventional cardiologists that routinely perform fractional flow reserve measurements may have noticed that an increase in the pressure gradient can be observed prior to adenosine administration, if contrast has just been injected. This is due to the contrast medium acting as a hyperaemic stimulus itself. However, the frame counting for CTFC ends early in injection and well before the dye reaches the microcirculation to develop contrast-induced hyperaemia. Nevertheless, preceding contrast injections combined with the temporary balloon occlusion during PCI may have caused downstream vasodilation. This would render a pseudo-hyperaemic state when the final fluoroscopic images were acquired, leading to a ‘falsely’ improved CTFC.

A second finding of interest is that patients on statins have a significantly higher coronary flow velocity after PCI compared with control patients who have not been taking statins. Earlier work with positron emission tomography imaging has shown that statin treatment improves myocardial perfusion [4]. However, in this study by Cerit and colleagues, the CTFC prior to PCI was statistically equivalent in the two groups suggesting a different mechanism. The improved CTFC after PCI is likely attributable to statin therapy protecting against periprocedural injury to the microcirculation related to manipulation of the atherosclerotic plaque. Indeed, the randomised ARMYDA study found that pre-treatment with atorvastatin for 7 days, resulted in significantly reduced rates of PCI-related myocardial injury in patients with stable ischaemic heart disease [4]. Given that Cerit and colleagues found serum lipid profiles to be similar in both groups, the improved CTFC after PCI is likely attributable to the pleiotropic properties of statins. The finding that C‑reactive protein levels were significantly decreased in patients on statins as compared with the control group, suggests a role for the anti-inflammatory properties of statins in the protection against periprocedural myocardial injury. The authors also discuss improvement of endothelium-dependent vasodilation mediated by statin as a possible reason for their findings. This appears less likely, given that no differences in CTFC between the statin and control group were observed prior to PCI.

Finally, a note of criticism is in order since CTFC has several inherent limitations. Firstly, as mentioned above, CTFC does not measure maximal hyperaemic flow and numerous factors, such as preceding saline or contrast injections, balloon occlusion and fluctuations in heart rate, may influence the resting state and CTFC. Secondly, although CTFC is a quantitative measure, it does partly depend on contrast injection speed [5]. Alternatives such as positron emission tomography imaging and invasive Doppler or thermodilution-based measurements provide more accurate estimations of flow. Thus, the use of CTFC is a clear limitation, even though this paper by Cerit and colleagues elegantly demonstrates potential beneficial pleiotropic effects of statins before elective PCI. Future studies will need to verify the current results involving dedicated flow measurements and using a randomised, propensity score matched or crossover design, before definitive conclusions can be drawn.
